# Neoantigen vaccine: an emerging tumor immunotherapy

**DOI:** 10.1186/s12943-019-1055-6

**Published:** 2019-08-23

**Authors:** Miao Peng, Yongzhen Mo, Yian Wang, Pan Wu, Yijie Zhang, Fang Xiong, Can Guo, Xu Wu, Yong Li, Xiaoling Li, Guiyuan Li, Wei Xiong, Zhaoyang Zeng

**Affiliations:** 1 NHC Key Laboratory of Carcinogenesis and Hunan Key Laboratory of Translational Radiation Oncology, Hunan Cancer Hospital and The Affiliated Cancer Hospital of Xiangya School of Medicine, Central South University, Changsha, Hunan China; 2 Key Laboratory of Carcinogenesis and Cancer Invasion of the Chinese Ministry of Education, Cancer Research Institute, Central South University, Changsha, Hunan China; 3 Hunan Key Laboratory of Nonresolving Inflammation and Cancer, Disease Genome Research Center, the Third Xiangya Hospital, Central South University, Changsha, Hunan China; 4 DEPARTMENT OF MEDICINE, Comprehensive Cancer Center Baylor College of Medicine, Alkek Building, RM N720, Houston, Texas USA

**Keywords:** Neoantigen, Tumor, Vaccine, Malignancy, Immunotherapy

## Abstract

Genetic instability of tumor cells often leads to the occurrence of a large number of mutations, and expression of non-synonymous mutations can produce tumor-specific antigens called neoantigens. Neoantigens are highly immunogenic as they are not expressed in normal tissues. They can activate CD4+ and CD8+ T cells to generate immune response and have the potential to become new targets of tumor immunotherapy. The development of bioinformatics technology has accelerated the identification of neoantigens. The combination of different algorithms to identify and predict the affinity of neoantigens to major histocompatibility complexes (MHCs) or the immunogenicity of neoantigens is mainly based on the whole-exome sequencing technology. Tumor vaccines targeting neoantigens mainly include nucleic acid, dendritic cell (DC)-based, tumor cell, and synthetic long peptide (SLP) vaccines. The combination with immune checkpoint inhibition therapy or radiotherapy and chemotherapy might achieve better therapeutic effects. Currently, several clinical trials have demonstrated the safety and efficacy of these vaccines. Further development of sequencing technologies and bioinformatics algorithms, as well as an improvement in our understanding of the mechanisms underlying tumor development, will expand the application of neoantigen vaccines in the future.

## Introduction

Malignant tumors are associated with high morbidity and mortality worldwide. According to the latest statistics released by GLOBOCAN, there were 18.1 million new cases of cancer and 9.6 million cancer-related deaths in 2018 [1]. Thus, malignant tumors constitute a considerable threat to human health [2, 3].

The traditional treatment for malignant tumors is based on surgery, radiotherapy, chemotherapy, and targeted treatment, each with its pros and cons. Surgery cannot always completely remove tumor cells, and recent studies suggest that reaction to post-operative wound healing may lead to the growth of metastatic tumors [4]. Radiotherapy and chemotherapy tend to elicit tolerance and recurrence of tumor cells, resulting in poor prognosis [5–8]. Specificity is the advantage of using targeted therapy. Early clinical trials on multiple tumor types have shown that single-molecule targeted therapy has a higher response rate and survival rate than other therapies [9–11], although problems such as unsatisfactory drug development and high cost persist [12].

In recent years, tumor immunotherapy has emerged as a new approach for eliminating malignant tumors. Checkpoints on the surface of T lymphocytes act as molecular brakes during immune response to maintain the balance of the immune system. Researchers have shown that tumor cells can express checkpoint inhibitors to achieve immune escape [13]. Allison and Honjo, winners of the Nobel Prize in physiology and medicine in 2018, showed that cytotoxic T lymphocyte antigen 4 (CTLA-4) and programmed cell death protein 1 (PD-1) act as negative immune regulatory factors and inhibit anti-tumor immune response. They also confirmed that antibody-mediated blockage of immune checkpoints removes the inhibition of immune cells by tumor cells and achieves anti-tumor effect [14–20], which forms the basis of the immune checkpoint inhibition therapy. Clinical trials have shown that immune checkpoint regulation therapy has good potential, although the effect is limited in many cases, especially in solid tumors where the response rate is low. Another popular immunotherapy is adoptive T-cell therapy, which is a type of passive immunotherapy. This method involves activation and amplification of the patient’s autologous T lymphocytes in vitro and then returning them to the body to kill tumor cells [21]. Currently, remarkable results have been achieved in clinical trials, although the effect on solid tumors is limited [22, 23]. However, adoptive T cells have poor in vivo persistence, cytotoxicity, and other defects [24, 25], and may trigger inflammatory factor storms.

In addition to immune system suppression, the weak immunogenicity of tumor cells is another reason underlying their immune escape. Therefore, the search for neoantigens with stronger immunogenicity has become a key issue in immunotherapy. Currently, sequencing technology and bioinformatics algorithm have made considerable progress, and researchers have clarified the role of major MHC proteins in antigen presentation [26, 27], realized the proliferation of antigen-specific T lymphocytes in vitro [28, 29], and cloned and expressed genes using molecular biological techniques. These advancements provide the necessary support for molecular identification of neoantigens. The presence of neoantigens is one of the essential differences between tumor cells and normal cells, and therefore, the concept of using the identified neoantigens as vaccines to actively stimulate patients’ autoimmune system and generate anti-tumor response has gained recognition. Theoretically, compared to other types of immunotherapy, the neoantigen vaccine, a new type of tumor immunotherapy, can induce strong specific immune response and elicit stable therapeutic effects.

This review will focus on the identification of neoantigens, designing of principles and clinical applications of neoantigen vaccines, and their combinations with other traditional or non-traditional antitumor therapies.

### Neoantigens

Neoantigens, which are non-autologous proteins with individual specificity, are generated by non-synonymous mutations in the tumor cell genome [30]. Owing to its strong immunogenicity and lack of expression in normal tissues, it is now an important target for tumor immunotherapy. Sixty years ago, Prehn et al. [31] proposed that tumor cells can express neoantigens from DNA with non-synonymous mutations. In the 1980s and 1990s, scientists hypothesized that tumor-specific antigens are present on the surface of tumor cells, which can be recognized and bound by patients’ human leukocyte antigen (HLA) molecules, thereby activating specific T cells and inducing anti-tumor immune responses [32]. However, the traditional cloning methods are expensive and cannot always accurately identify tumor neoantigens, which limits the application and development of neoantigens as tumor vaccines. The rapid development of high-throughput sequencing technology, including whole-genome sequencing and the whole-exon sequencing, which are now less expensive and more convenient than they have been in the past, has led to explosion of sequencing data and identification of thousands of tumor-associated genes. Mutations affecting the process of tumorigenesis and development have also been identified [33–35], and studies are focusing on neoantigens that can be specifically recognized by T cells.

Neoantigens, a class of tumor-specific antigens, differ from the traditional tumor-associated antigen (TAA). TAA is not unique to tumor tissue as it is also present in normal tissues; it is highly expressed in proliferating tumor cells expressing HER2, MART-1, MUC1, and MAGE [36]. However, in vivo experiments by Prehn et al. [31] showed that antigens that elicit strong tumor rejection tend to exhibit strong individual specificity. Therefore, compared to TAAs, neoantigens possess stronger immunogenicity and higher affinity toward MHC, and are not affected by central immunological tolerance. Using an ultraviolet light-induced mouse tumor model, Monach et al. [37] showed for the first time that tumor neoantigens can be targeted for cancer immunotherapy. The larger the difference between mutation sequence and original coding sequence, the more obvious the “non-self” feature of the abnormal protein and stronger the immunogenicity. Point mutations account for 95% mutations in tumors, whereas insertion-deletions (indels) and frame-shift mutations account for the rest [38, 39]. The amino acid sequence and spatial structure changes caused by indel or frame-shift mutations were more obvious, and the mutant peptide had a stronger affinity to MHC and was more likely to be recognized as a neoantigen by T cells [40]. However, due to the poor immunogenicity of a variety of tumors, or the decline of patients’ autoimmune system function, the proportion of T cells spontaneously recognizing endogenous neoantigens is about 1–2% [41], therefore designing specific vaccines on the basis of obtaining efficient neoantigens will be an effective tumor immunotherapy.

### Identification and prediction of neoantigens

Neoantigens are highly individual-specific and usually do not involve known oncogenes. Hence, identification of neoantigens is critical for tumor vaccine therapy. Sequencing depth, quality of tumor tissue, source of the sequencing material, single nucleotide variants (SNV) algorithm, and other factors affect neoantigen identification [42–44]. The first step in neoantigen recognition is often the rapid comparison of the DNA sequences of tumor cells and normal cells using high-throughput sequencing techniques. As mutations in tumor cells are complex and include non-coding mutations and nonsense mutations, expression and screening of mutant proteins from these mutated DNA sequences are challenging [45]. With the development of sequencing technology and bioinformatics algorithms, the accuracy and reliability with which neoantigens can be predicted and identified have increased. The whole-exon sequencing technology can identify neoantigens with high efficiency, wide coverage, and low false negative rate. Currently, the majority of neoantigens are identified using the whole-exon sequencing technology [46].

Whether mutations can form tumor neoantigens depends on several factors: 1) whether the mutated sequence can be translated into protein; 2) whether the mutated protein can be processed into peptides and presented; 3) affinity between the mutated peptide and MHC molecules of the patients; 4) affinity of mutant peptide-MHC complex with T cell receptor (TCR) [47]. Therefore, the prediction of neoantigens requires not only identification of genome-expressed mutations, but also data regarding patients’ MHC types. Currently, various types of software applications are being used for the identification of neoantigens [48–51], and some commonly used software packages are listed in Table 1.

**Table 1:** The summary of neoantigen prediction software

Software	Principle	Year
HLAminer [52]	Based on the shotgun sequencing database from Illumina platform, the HLA type was predicted by orienting the assembly of shotgun sequence data and comparing it with the reference allele sequence database	2012
VariantEffect Predictor Tool [53]	Automate annotations in a standard way to reduce manual review time, annotate and prioritize variants	2016
NetMHCpan [54]	Sequence comparison method based on artificial neural network, and predict the affinity of peptide-MHC-I type molecular	2016
UCSC browser [55]	Based on sequence search, the fusion of multiple databases can provide fast and accurate access to any gene segment	2002
CloudNeo pipeline [56]	Docker container was used to complete the tasks in the workflow. After the mutant VCF file and bam file representing HLA typing were input respectively, the HLA affinity prediction of all mutant peptides was obtained	2017
OptiType [57]	The HLA typing algorithm based on integer linear programming provides sequencing databases including RNA, exome and whole genome	2014
ATHLATES [58]	Assembly, allele recognition and allele pair inference were applied to short sequences, and the HLA genotyping at allele level was achieved by exon sequencing	2013
pVAC-Seq [59]	To integrate tumor mutation and expression data and identify personalized mutagens by tumor sequencing	2016
MuPeXI [60]	The extraction and induction of mutant peptides can roughly identify tumor-specific peptides, predict their immunogenicity, and evaluate their potential for new epitopes	2017
Strelka [61]	Based on a new Bayesian model, the matching tumor-normal sample sequencing data was used to analyze and predict somatic cell variation, with high accuracy and sensitivity	2012
Strelka2 [62]	Based on the mixed model, the error parameters of each sample insertion or deletion were estimated, and the liquid tumor analysis was improved	2018
VarScan2 [63]	Somatic and copy number mutations in tumor-normal exome data were detected by heuristic statistical algorithm	2012
Somaticseq [64]	Based on a randomized enhancement algorithm, more than 70 individual genome and sequencing features were extracted for each candidate site to accurately detect somatic mutations	2015
SMMPMBEC [65]	Using matrix as a Bayesian prior, based on the optimal neural network predicting peptide with MHC-I type molecules	2009
NeoPredPipe [66]	Based on a pipeline connecting commonly used bioinformatic software via custom python scripts to provide neoantigen burden, tumor heterogeneity, immune stimulation potential and HLA haplotype of patients	2019

In the process of defining the specificity of the anti-tumor immune response, MHC-II type molecules present antigens, which are recognized by CD4 + T cells. However, owing to the uniqueness of MHC-II structure and the complexity of the process via which peptides combine with MHC-II molecules, powerful and abundant databases on these interactions are lacking [67], Therefore, further development of bioinformatics is required to improve identification and evaluation of neoantigens.

### Principle of neoantigen vaccines

Unlike common prophylactic vaccines, tumor vaccines are administered to patients with malignant tumors, supplemented by appropriate adjuvants, to activate the patient’s autoimmune response and kill the tumor cells [68–70]. Mutations in tumor cells change the amino acid sequences of proteins, which are then translated and processed into short peptides [71] called tumor neoantigens. As non-autoantigens, neoantigens are exposed to MHC molecules, which subsequently trigger the body’s antitumor immune response.

In 1891, Dr. William Coley, the pioneer of tumor immunotherapy, used Coley’s toxin (inactivated *Streptococcus pyogenes* and *Serratia marcescens*) for intratumoral injection to stimulate the patient’s immune system, following which, occasional continuous tumor regression was observed [72]. Kugler et al. [73] fused tumor cells with dendritic cells using electrofusion technology; the fused cells not only expressed the tumor antigen, but also possessed the co-stimulation ability of dendritic cells. In patients with renal cancer, the fusion cells induced proliferation of autologous T lymphocytes and differentiation of cytotoxic lymphocytes (CTLs). Owing to technological limitations, the design of earlier tumor vaccines was relatively simple, and it was difficult to accurately locate the immunological target. Despite a certain degree of anti-tumor effect, the results were far from expected.

The therapeutic effect of tumor vaccines often depends on the difference in the expression of the targeted antigen between tumor cells and normal cells. As foreign antigens, neoantigens can not only enhance the anti-tumor immune response, but also reduce the risk of autoimmunity. Hence, neoantigen-activated T cells can produce highly active T cells, TCRs of which show stronger affinity toward MHC-neoantigen-peptide complexes and avoid clearance by central immune tolerance [74]. Among the non-synonymous mutations in the genome of cancer cells, driver mutations are special as they provide selective growth advantages for cancer cells. Compared to non-driver mutations, driver mutations have an obvious clonal tendency [75] and are possibly present in all cells of tumor tissues. Schumacher et al. [76] observed that accumulation of monoallelic point mutations in isocitrate dehydrogenase type 1 (IDH1) is an early and decisive event in the development of glioma subsets and other types of tumors, which can lead to the occurrence of new enzyme functions, genome hypermethylation, production of the oncogenic metabolite 2-hydroxyglutarate (2-HG), genetic instability, and malignant transformation of cells [77–79]. The IDH1 peptide was used to vaccinate mice, which triggered an MHC-II type effective and restrictive anti-tumor immune response. Owing to the rapid development of sequencing technology and the continuous optimization of bioinformatics algorithms, researchers can now accurately identify tumor neoantigens and predict their MHC affinity and immunogenicity, resulting in the development of personalized medicine. Based on the definition of neoantigens or driving antigens, various types of cancer cell vaccines have been designed, including tumor cell vaccine [80], long peptide vaccine or protein vaccine [81, 82], genomic vaccine [83], and DC-based vaccine [84, 85].

**Fig. 1:**
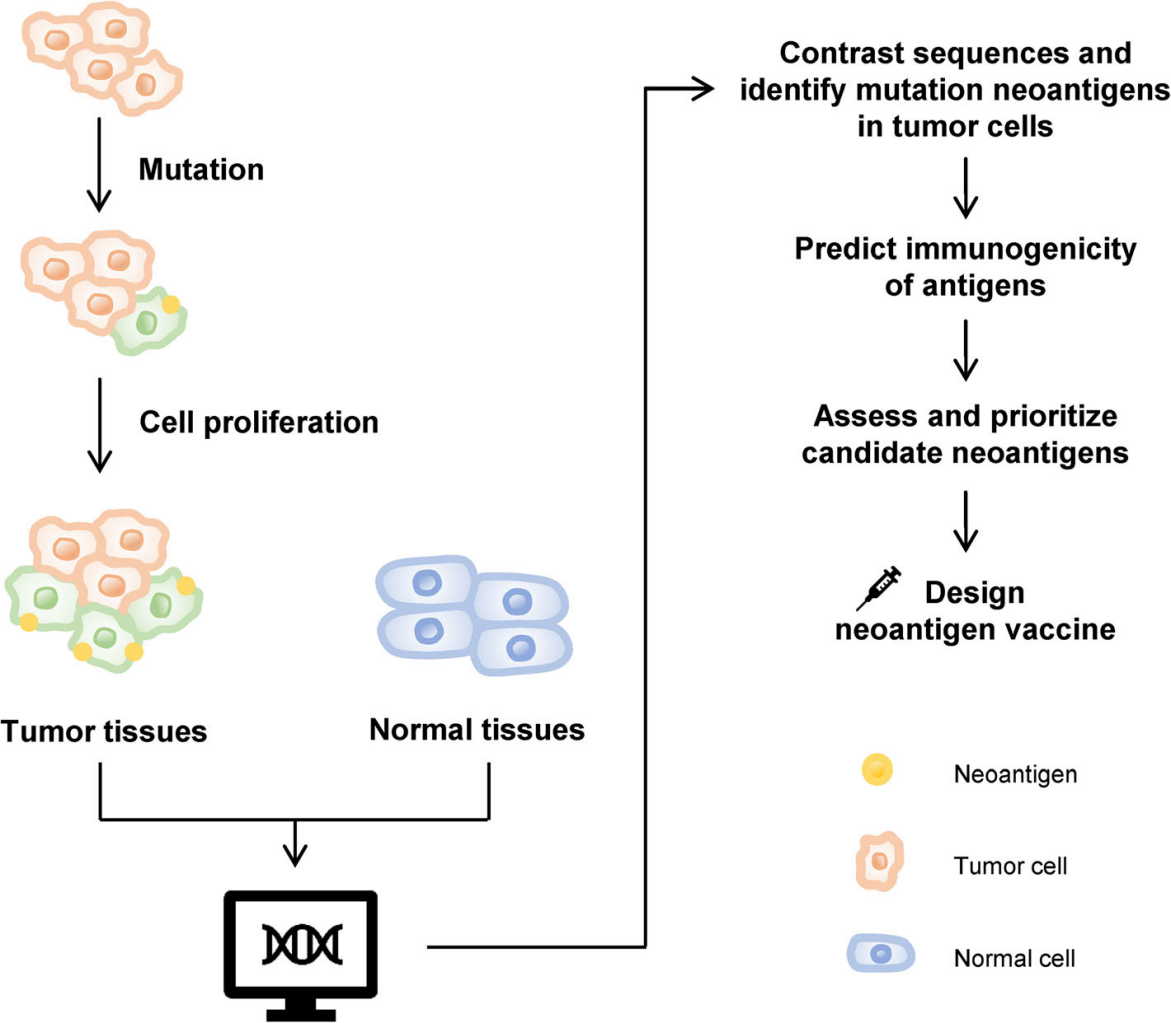
Mutations in tumor tissue produce neoantigens. Clonal neoantigens can be expressed by a large number of proliferating tumor cells. Various software packages were used to compare the sequence differences between tumor cells and normal cells, and to predict and prioritize the immunogenicity of antigens for screening the optimal tumor neoantigens

With the optimization of the prediction algorithm of immunogenicity, research on tumor vaccines targeting neoantigens has progressed rapidly, and hopefully neoantigen vaccines will soon completely replace tumor vaccines targeting shared TAAs (Fig. 1).

### Clinical progress

Traditional tumor vaccines mainly target TAAs, which are shared between tumor cells and normal cells [86]. Owing to the presence of central immunological tolerance in the thymus, the active T cells that recognize TAA or other autoantigens are likely to be eliminated during development, which affects the efficacy of tumor-targeted vaccines [87, 88]. Several clinical trials targeting TAAs have shown that long-term therapeutic effects are difficult to achieve with anti-tumor vaccines [86]. P1A is the first recognized non-mutated tumor-related antigen. Sarma et al. [89] developed transgenic mice that can express P1A-specific receptor on the surface of all T cells. For P1A-expressing tumor cells, T cells were unable to produce sufficiently strong killing effect.

Current genomics and bioinformatics technologies can identify tumor-specific missense mutant proteins that act as tumor neoantigens in tumor vaccines [90]. Several clinical trials have shown that neoantigens can be recognized by CD8+ and CD4 + T cells in tumor tissue, and thus trigger an anti-tumor immune response in vivo [91, 92]. Castle et al. [93] used SLPs derived from 50 effective mutations to immunize B16F0 mouse melanoma models. Results showed that neoantigen peptide vaccines targeting MUT30 and MUT44, two mutated antigens, had significant preventive and therapeutic effects in mouse tumor models.

Carreno et al. [94] was the first to report that DCs loaded with neoantigens can trigger specific T cell responses in patients with melanoma. In this study, whole-exon sequencing, computer-simulated epitope prediction, and immunohistochemistry were used to identify neoantigens on tumor cells. This was also the first study to show that the antigen can be identified by CTLs in three patients with melanoma. Subsequently, DCs loaded with neoantigens were cultured in vitro for autologous transfusion. Results showed that the DC-based neoantigen-specific vaccine triggered neoantigen-specific T cell response that was not detected before injection and enhanced the existing immune response. Of the three patients with melanoma, two were stable, and one showed no side effects or recurrence.

The RNA neoantigen vaccine has unique advantages. When adequate tumor tissue is not available, RNA extracted from a small number of cancer cells is amplified for vaccine preparation. Compared to DNA vaccines, RNA vaccines can avoid integration into host cell genome and avoid potential risks. Sahin et al. [50] was the first to identify neoantigens using the next generation sequencing (NGS) database, and prepared RNA vaccines capable of encoding neoantigens using computer simulation and predictive binding. These RNA molecules were previously shown to be captured by DCs in lymph nodes [95]. In total, 13 patients with melanoma received the RNA vaccine, eight of whom had no tumor development during follow-up. Immunosurveillance analysis of peripheral blood mononuclear cells (PBMCs) in patients showed that RNA vaccines can enhance the existing neoantigen-specific T cell response and induce new T cell response. Ott et al. [49] identified neoantigens and used bioinformatics algorithm to predict the combination of neoantigens and MHC-I molecules; the prepared SLP vaccine was injected in six patients with surgical resection of the tumor. Results showed that the tumor did not recur in four patients in the 32 months after inoculation.

In a recent study, a neoantigen vaccine was shown to affect glioblastoma, which lacks T cell infiltration and has low mutation rate. Hilf et al. [96] prepared two highly personalized vaccines and inoculated 15 patients with HLA-A*02:01- or HLA-A*24:02-positive glioblastoma, which elicited continuous T cell response and improved patients’ median total survival time to 29.0 months. Keskin et al. [97] administered neoantigen vaccine to glioblastoma patients after surgical resection and conventional radiotherapy and observed that the vaccine activated specific T cells, which migrated from the peripheral blood into the brain, changing the immune environment of glioblastoma.

In addition, clinical trials have shown that for patients with early-stage tumors, the tumor vaccine is more effective, while for patients with late-stage tumors, it is less effective than expected. Hanna et al. [98] conducted three multi-mechanism, prospective, randomized, controlled clinical trials to assess therapeutic efficacy in patients with stage II and III colon cancer after surgical removal of tumor and injection of a tumor vaccine. They monitored four parameters, namely recurrence time, total survival period, disease-free survival period, and survival without recurrence. Results showed that the disease-free survival period of patients receiving tumor vaccine was longer than those in the control group, and the effect of the treatment on patients with stage II disease was significantly better than that on patients with stage III disease. Therefore, timely treatment with tumor vaccine can yield results closer to the expected effect.

**Fig. 2:**
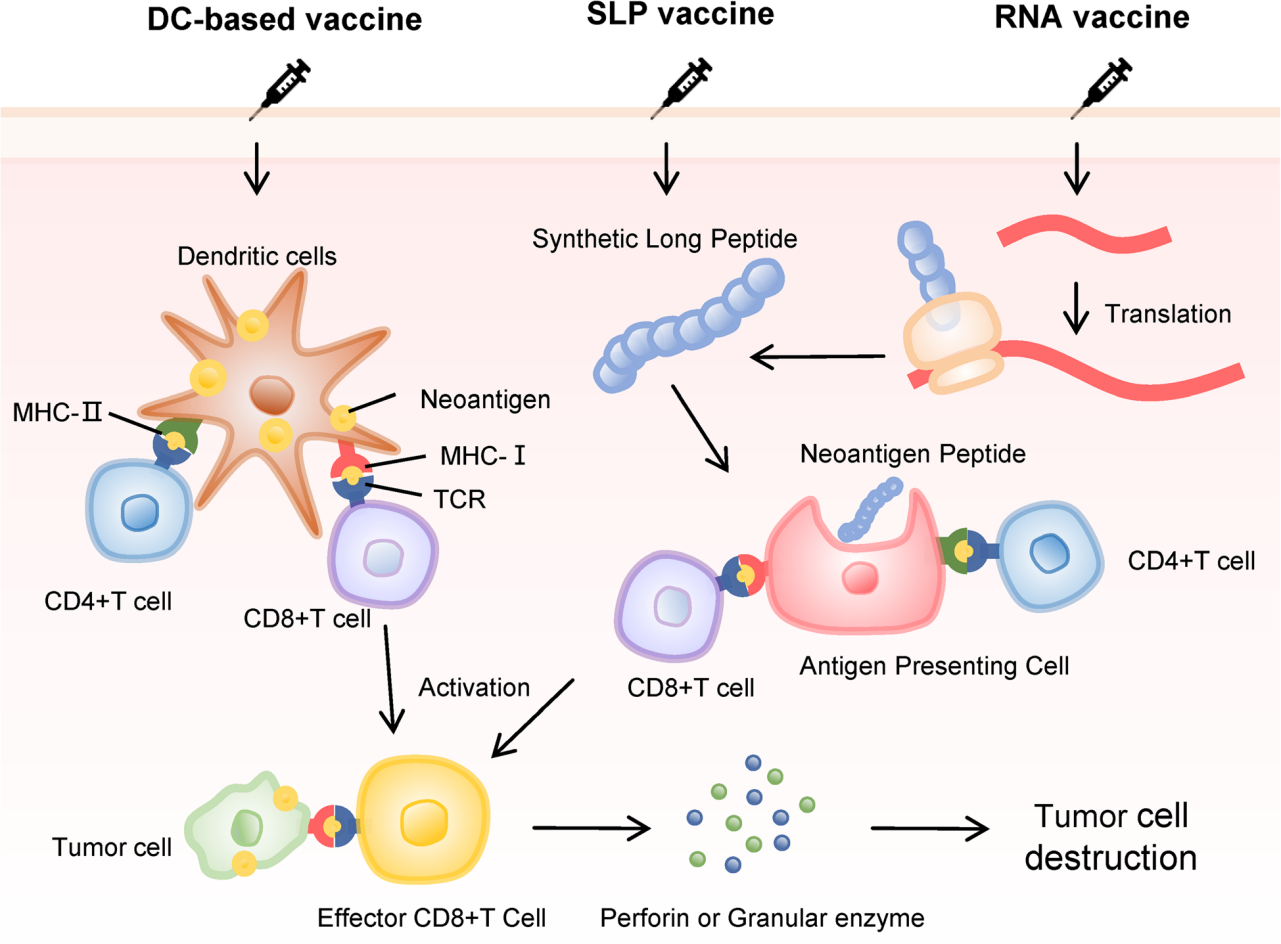
Major types of neoantigen vaccine. In vivo, neoantigens are eventually presented to CD4+ T cells and CD8+ T cells to induce specific immune responses and achieve anti-tumor effects

Overall, RNA-, SLP-, DC-based vaccines, and other neoantigen vaccines have been tested in strict phase I clinical trials, and the results were in agreement with the expected results. These preliminary results indicated that neoantigen vaccines based on DCs, SLP, and RNA are safe and have the ability to induce CD8+ and CD4+ specific T cell responses, highlighting the considerable potential of this immunotherapy (Fig. 2).

### Combination of neoantigen vaccine with other therapies

Although neoantigen vaccines can stimulate autoimmune response, tumor cells possess various immune escape mechanisms; in addition, the tumor microenvironment also interferes in the function of immune cells, and even inhibits immune response [99–106], which impedes the vaccine from exhibiting its optimal effect in vivo. Yadav et al. [51] used mutated DPAGT1, REPS1, and ADPGK to prepare peptide vaccines that can delay the growth of tumor cells in mouse models; at the same time, they observed that neoantigen-specific T cells expressed high levels of PD-1 and TIM3 receptors, which acted as negative regulators of immune response, and even induced apoptosis of T cells, suggesting that T cells become dysfunctional during this process [107]. Therefore, combination of neoantigen vaccine and other therapies is required to achieve the expected effect of the vaccine.

Tumor vaccines stimulate the patients’ immune system, especially the response of specific CD8+ T cells [68]; however, interferon gamma (IFNγ) produced by CD8+ and Th1 CD4+ cells regulate the expression of PD-L1 [108–110]. PD-L1 expression in tumor cells is upregulated when attacked by T cells [111]. Therefore, tumor vaccines induce the production of specific T cells and simultaneously upregulate the expression of PD-L1, inhibiting the function of tumor vaccines [112, 113]. In addition, while the immune system is activated, the expression of T cell surface reporter CTLA-4 is correspondingly increased, which binds with the ligand B7–1/B7–2 on Antigen-presenting cells and plays an immunosuppressant effect. Checkpoint inhibition therapy involves the use of specific monoclonal antibodies, namely, anti-CTLA-4 [114], anti-PD-1 [115], and anti-PD-L1 antibodies [116], which bind to the immune checkpoint proteins of T cells to remove the inhibition of T cell function by tumor cells [117]. This therapy has a lasting clinical effect and is effective for patients with multiple malignant tumors [118], although patients lacking tumor-specific effector T cells do not respond to immune checkpoint inhibition therapy [119–121].

Comparative studies have shown that the combination of tumor vaccine and immunosuppressive therapy is more effective than monotherapy [122, 123]. Curran et al. [124] designed mouse models to show that the vaccine secreting granulocyte/macrophage-colony stimulating factor (GM-CSF) or Flt3-ligand, combined with PD-1 and CTLA-4 blocking therapy, can effectively prolong the survival period and improve the ratio of effector cells to regulatory cells in the tumor microenvironment of mice. Ott et al. [49] reported that six surgically resected patients with melanoma were injected with synthetic neoantigen peptides. Two of them had poor therapeutic effects and achieved complete anti-tumor immune responses after treatment with the PD-1 antibody.

Combinations of neoantigen vaccine and adaptive T cell therapy have also been successfully used to achieve anti-tumor response. Matthias et al. [125] reported that mutation-specific TCR might provide efficient anti-tumor response under appropriate condition. The team used ultraviolet radiation to generate the mouse tumor model and divided the tumor tissue into 20 fragments, followed by analysis of the antigenic composition of different parts, and finally obtained the main neoantigens existing in all the 20 tumor tissue blocks [126]. The antigen is called mp68 and Matthias’s team designed T cells that express a high-affinity mp68 TCR, which was administered to mice. Results showed that the therapy can destroy intratumorous blood vessels and destroy larger, longer-lived solid tumors. Using full excision sequencing, Tran et al. [127] demonstrated that the lymphocyte infiltrate of tumors from patients with metastatic cholangiocarcinoma contained CD4+ T helper 1 (Th1) cells, which can identify a mutated HLA-II antigen from erbb2 interacting protein (ERBB2IP) in the carcinoma. After the incorporation of tumor infiltrating lymphocytes (TILs) containing ~ 25% neoantigen-specific auxiliary Th1 cells into tumor tissues, the target lesions of the patients reduced, and the stable time of the disease was prolonged, leading to significant tumor regression. In the current study, Song et al. [128] sequenced whole exome and transcriptomes in patients with epithelial ovarian cancer (EOC) to identify neoantigen candidates and then analyzed the reactions of neoantigen-specific CD4+ and CD8+ T cell response in tumor or the peripheral blood. The specific T cell receptors (TCR) were transferred to peripheral blood T cells, making them with a neoantigen reactivity. It is another feasible strategy to eventually achieve the personalized trans-T cells transfer immunotherapy.

However, CAR-T therapy has limited efficacy due to the fact that CAR-T cells transfer into the patient through intravenous injection, as the blood circulates to the tumor site, T cells could identify neoantigens and be activated, while the microenvironment of solid tumors blocks CAR-T cells. Ma et al. [129] designed amphiphilic ligands (amph-ligands) in the latest study, which effectively alleviated this problem. The head of amph-ligands contain antigens to activate the CAR-T cells, at the other end, amph-ligands are equipped with long tail of lipids, which binds to free albumin in the blood and rapidly arrives at lymph nodes to join CAR-T cells. Firstly, the researchers demonstrated that this type of amph-ligands vaccine can dose-dependent activate CAR-T cells to exert tumor killing effect in vitro. Then they inoculated amph-pepvIII vaccine and EGFRvIII CAR-T cells in glioma mice, observed significant amplification and intratumoral infiltration of CAR-T cells in peripheral blood. Subsequent experiments in various mouse tumor models eventually achieved the complete elimination of 60% of mouse tumors, showing the great potential of the combination of neoantigen and CAR-T therapy.

Several factors can lead to immune dysfunction in the tumor microenvironment, such as T-Regulatory cells (Tregs), myeloid-derived suppressor cells (MDSCs), potassium, and immunosuppressive DC cells that inhibit the activity of T cells [130]. Spranger et al. [109] showed that various immunosuppressive factors are immune-intrinsic. After the infiltration of effector T cells, FoxP3+ Tregs is recruited at tumor sites as a negative feedback regulatory mechanism driven by CCR4-binding chemokine along with induced proliferation component, indicating that tumor vaccines could induce effector T cells and increase Tregs population in the tumor at the same time. Klages et al. [131] observed that tumor growth was retarded when a transgenic diphtheria toxin receptor was used to prostrate Tregs in a mouse model of melanoma, thereby significantly improving the anti-cancer effect of the tumor vaccine. Other two studies conducted by Casares et al. [132, 133] showed that FoxP3 inhibitory peptide P60 occupied the intermediate domain of FoxP3, inhibited its homologous dimerization and binding with transcription factors, attenuated the activity of Tregs in vivo and in vitro, and enhanced the efficacy of tumor vaccines in mouse models.

In addition, tumor necrosis can inhibit the activity of anti-tumor T cells. After tumor cell necrosis, intracellular potassium ions are released into the extracellular space and are enriched in tumor-specific effector T cells, which can inhibit the activity of Akt protein kinase, enhance the inhibition of potassium ion-induced T cell function, and prompt immune escape of tumor cells [134]. Inhibition of this potassium-induced immune suppression, combined with a tumor vaccine, can enhance the killing effect of tumor-specific T cells.

Traditional treatments such as radiotherapy and chemotherapy can also enhance the role of neoantigen vaccines. Studies have shown that chemotherapy or radiotherapy can induce tumor cells to release more antigens. The combination of neoantigen vaccine and chemoradiotherapy can circumvent this problem when the number of tumors neoantigens is too low to activate T-cell response [135]. Radiotherapy can also enhance the transport of T cells into tumor tissue and increase the intensity of specific anti-tumor immune response [136]. In addition, several reports have shown that certain chemotherapeutic drugs can enhance the anti-tumor activity of adoptive T cells [137, 138], macrophages [139], and tumor vaccines [140, 141]. For example, pretreatment with CTX (cyclophosphamide) and other preparations, followed by tumor vaccine injection, can enhance the number and functional activity of neoantigen-specific T cells. The optimal immunomodulatory dose is reached when the dose of the chemotherapeutic drug is higher than the dose at which cytopenia is induced [142]. In addition, chemoradiotherapy can reduce immune suppression in the tumor microenvironment [143]. Although application of radiotherapy and chemotherapy alone cannot completely eliminate large numbers of tumor cells, combination with neoantigen vaccines shows considerable prospects. Immunologically-mediated and radiation-driven personalized systemic therapy model [144] is also a new concept in the field of personalized therapy.

**Fig. 3:**
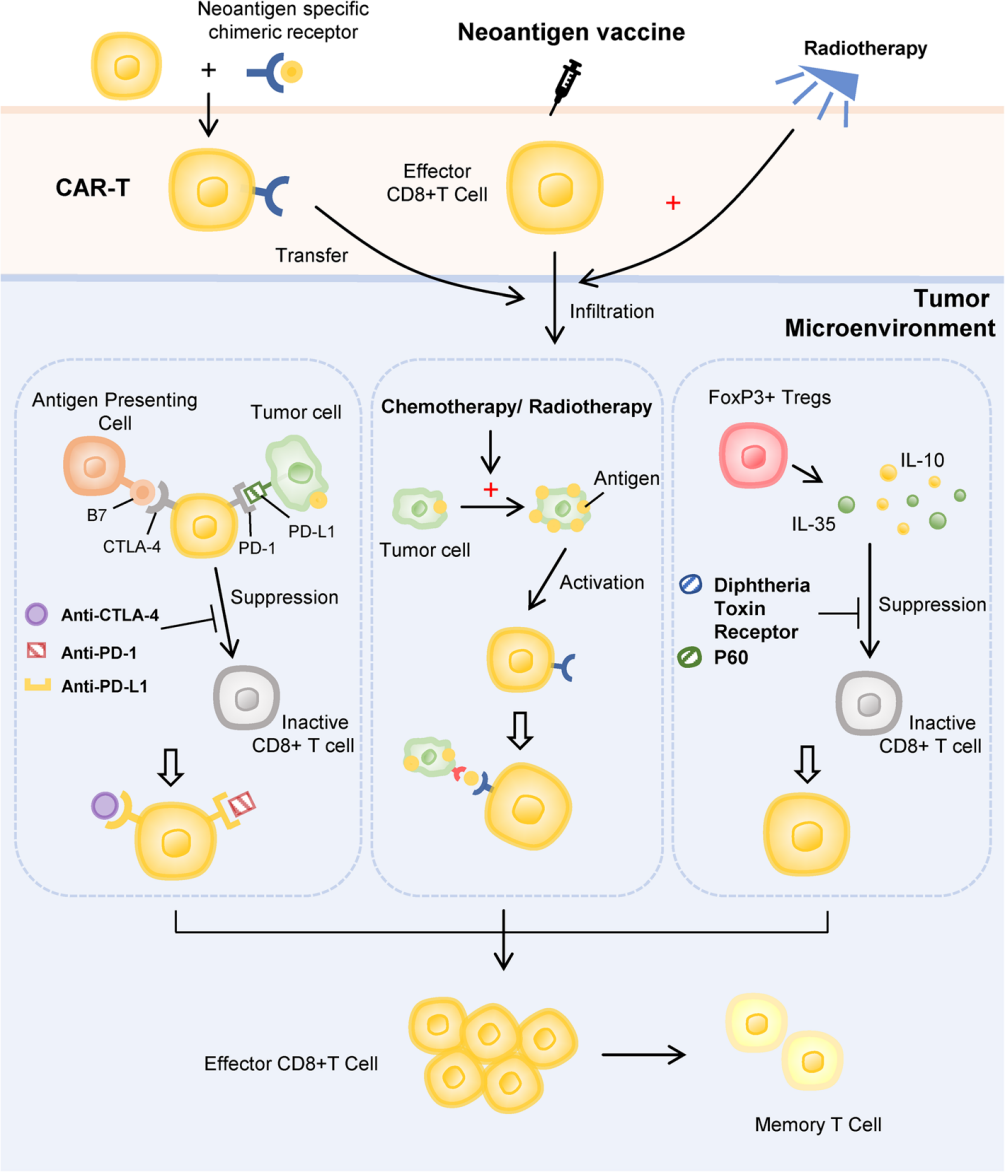
Combination of neoantigen vaccines with other therapies. Combination of neoantigen vaccines with the checkpoint inhibition therapy can relieve the tumor cell-mediated inhibition of effector T cells. Radiotherapy and chemotherapy can assist vaccines play a better effect. Drugs targeting the immunosuppressive factors in the tumor microenvironment were administered to circumvent the inactivation of T cells by various molecules and cells in the tumor microenvironment. In combination with CAR-T therapy, T cells specifically targeting neoantigens were cultured in vitro and then injected into the body to generate effector T cells and memory T cells, thereby enhancing the anti-tumor effect

Compared to traditional therapy and vaccines based on shared antigens, neoantigen vaccine has obvious advantages and lower side effect; however, effective and long-term therapeutic effects are not observed when it is used for monotherapy. Nonetheless, combined with other methods, such as immune checkpoint inhibition and immune inhibition in the tumor microenvironment, it can produce stronger antitumor response [49] (Fig. 3).

### Disadvantages and future directions

Neoantigen vaccines alone cannot achieve complete elimination of malignant tumors. Melief et al. [145] postulated that insufficient maturity of the selected and recognized neoantigens is one of the reasons why neoantigen vaccines cannot completely eradicate tumors. The occurrence and development of tumor is a dynamic evolutionary process characterized by genetic instability. Many types of mutations are generated, cloned, altered, and lost from the tumor cell genome. Recent technology allows analysis of the genomes of single tumor samples collected at specific time points, which, however, does not provide information regarding the heterogeneity in tumors [146, 147]. Vaccines can only kill a small number of tumor cells if the neoantigens targeted by the vaccine are derived from mutated subclones, which restricts clinical effect [148]. As driver mutations possibly exist in all cells within a certain tumor, designing of vaccines targeting these neoantigens is important; however, it is often difficult to translate a mutation to a neoantigen. In melanoma, only about 8% neoantigens are derived from driver mutations, and 92% are from non-driver mutations [47]. Methods of identifying effective and common neoantigens and improving activation of immune cells are challenges for tumor vaccine designing.

The use of neoantigen vaccines is also limited by the diversity of somatic mutations in different tumor types and their individual specificity. Studies have shown that the immune activity correlated positively with the tumor mutation burden (TMB) of tumors [149]. Only 10% of the non-synonymous mutations in tumor cells can generate mutant peptides with high MHC affinity [40], while only 1% peptides with high MHC affinity can be recognized by T cells in patients [150]. Therefore, theoretically, the higher the TMB, the more neoantigens can be recognized by T cells in tumors. The TMB varies considerably with different types of malignant tumors. Tumors with high TMB, such as melanoma, have a higher response rate to immunotherapy, whereas tumors with generally low TMB are not suitable for the existing neoantigen vaccine system. Common chromosomal abnormalities in nasopharyngeal carcinoma are predominant in southeast Asia and some other regions, but the mutation rate is lower than those of other types of malignant tumors; furthermore, the median rate of somatic cell mutation per megabase is one [151], indicating that identification of neoantigens in the tumor tissues of nasopharyngeal carcinoma is challenging. Owing to significant differences in mutations among the three subtypes of nasopharyngeal carcinoma [152], the TMBs varied and the neoantigens produced were not identical.

But this is not always the case, as pediatric tumors often show significantly fewer somatic cell mutations, Zamora et al. [153] have shown that although children with acute lymphoblastic leukemia TMB is lower, but still could be induced strong antitumor immune response, which suggests the hematological malignancy may have better immunogenicity. Parkhurst et al. [154] performed high-throughput immunoscreening and whole exome sequencing of 75 patients with common gastrointestinal tumors, and identified 124 neoantigen-reactive tumor-immersed lymphocyte populations, which showed that even though some common epithelial tumors were considered to be low-immunogenicity, they also had the function of activating immune recognition and offered the possibility of immunotherapy on some extent.

Moreover, the neoantigens produced by each tumor are almost specific even for the same tumor type and there is no sharing between patients [155], and the probability of different individuals developing the same neoantigens is extremely low [156]. Therefore, neoantigens must be searched based on tumor types. Thus, large differences among tumor types and individuals limit the use of tumor vaccines targeting mutated neoantigens.

Owing to the limitation of the whole-exon sequencing technology, previous neoantigen assays are often limited to 2% of the coding sequence of the human genome. Recently, Perreault et al. developed a new protein genomics approach for analyzing non-coding regions, and their results showed that any type of non-coding region can produce abundant aberrantly expressed tumor-specific antigens (aeTSAs), a small part of which is generated by the mutation, whereas the majority arises from epigenetic changes in atypical translation events. The number of aeTSAs exceeds that of the neoantigen produced by mutations in coding regions [157]. Unlike the highly individual specificity of mutated neoantigens, aeTSAs can be shared by multiple individuals with tumors [158, 159]. Identification of more efficient neoantigens and aeTSAs in non-coding regions was a breakthrough in the field of tumor vaccines.

Immune escape of tumor cells is a critical issue impeding the efficacy of tumor vaccines. Tran et al. [160] observed loss of heterozygosity of chromosome 6, which encodes HLA-C*08:02, in 4095 patients, and showed that this molecule is essential for adoptive KRAS G12D-specific T cells to recognize tumors, thereby providing direct evidence regarding immune escape of tumor cells. Loss of heterozygosity of the HLA site limits clinical responses to tumor vaccines targeting neoantigens or adoptive T cell therapies. In addition to antigen loss, tumor cells possess various complex immune escape mechanisms, including suppression of immune checkpoints such as PD-1 and CTLA-4, immunosuppressive effect of various cells in the tumor microenvironment [161], and release of ions or proteins inside tumor cells after necrosis, all of which compromise the recognition of neoantigens by T cells and their activation. Neoantigen vaccine combined with other immunotherapies can prevent partial immune escape; however, many mechanisms are yet to be clearly elucidated, which impedes the clinical application of neoantigen vaccines. At the same time, the combination of tumor vaccines with other traditional therapies, such as chemoradiotherapy and targeted therapy, show immense potential for development. In addition, Muhammad et al. [162] adopted a different strategy for this problem recently. They transfected monocyte derived DC with neoantigen encoding mRNA to prime autologous naive CD8+ T cells in healthy donors. This program makes the activation of T-cells unaffected by the immunosuppressive environment of tumor hosts, which provides a new idea for us.

Although the current sequencing technology has triggered rapid development [45], identification and verification of neoantigens are still time-consuming and expensive, and the process of preparing vaccines from tissue samples usually takes 3–5 months [49, 50]. This long preparation period seriously limits the clinical application of neoantigen vaccines. Problems such as the high demand for tumor tissue in the identification process and the low yield of peptides after immunoaffinity purification are the technical obstacles that are currently difficult to overcome [163]. There is still room for further optimization of the neoantigen prediction algorithm. In addition to predicting the combination of different MHC molecules with neoantigens, it is necessary to predict potential neoantigens generated by gene fusion, indels, and other changes.

## Conclusion

Neoantigens are mutated antigens specifically expressed by tumor tissue and are not expressed on the surface of normal cells. Development of sequencing technology has improved the accuracy of identification and localization of neoantigens. Neoantigens are highly specific for individuals, and hence, tumor vaccines targeting neoantigens can effectively induce tumor-specific T cells in patients without killing normal cells, thereby achieving personalized precision treatment. As an emerging anti-tumor immunotherapy, neoantigen vaccine has achieved the expected therapeutic effect in several trials, improving the quality of patients’ lives to a certain extent and extending the survival period. SLP-, RNA-, DNA-, and DC-based vaccines, as well as other types of vaccines, have shown excellent safety and induction ability. The development of bioinformatics will further improve the recognition and identification of neoantigens.

Owing to the immune escape mechanism of tumor cells, neoantigen vaccines may not be able to exert their expected killing effect after inducing specific T cells, which is also one of the limitations regarding the application of tumor vaccines. Changes occur constantly during tumorigenesis and development, which enables tumors to survive in the complex immune environment. Further in-depth understanding of oncology and tumor immunology, and elucidation of the immune suppression and escape mechanisms of tumor tissue, will aid in developing more effective strategies.

## Data Availability

Not applicable.

## Abbreviations

2-HG: 2-hydroxyglutarate
aeTSA: Aberrantly expressed tumor-specific antigen
CTL: Cytotoxic lymphocyte
CTLA-4: cytotoxic T lymphocyte antigen 4
CTX: Cyclophosphamide
DC: Dendritic cell
GM-CSF: Granulocyte/macrophage-colony stimulating factor
HLA: Human leukocyte antigen
IDH1: Isocitrate dehydrogenase type 1
IFNγ: Interferon gamma
Indels: Insertion-deletions
MDSC: Myeloid-derived suppressor cell
MHC: Major histocompatibility complex
NGS: Next generation sequencing
PBMC: Peripheral blood mononuclear cell
PD-1: Programmed cell death protein 1
SLP: Synthetic long peptide
SNV: Single nucleotide variant
TAA: Tumor-associated antigen
TCR: T cell receptor
TIL: Tumor infiltrating lymphocyte
TMB: Tumor mutation burden
Treg: T-Regulatory cell
